# Predicting Pathologic Bone Lesions Using Scout Computed Tomography (CT) Imaging

**DOI:** 10.1155/2020/5105196

**Published:** 2020-08-06

**Authors:** Michael J. Colello, Erin R. Pichiotino, Stephanie L. Tanner, Scott E. Porter, Richard W. Gurich

**Affiliations:** Department of Orthopedic Surgery, Prisma Health–Upstate, Greenville, SC 29605, USA

## Abstract

The purpose of this study is to evaluate the benefit of reviewing scout CT images, obtained for routine oncologic surveillance, for the early identification of pathologic bony lesions. A retrospective review was conducted on patients who previously underwent surgical treatment by two orthopedic oncology surgeons at a tertiary care institution from 2009–2019 for pathologic lesions or fractures of the humerus or femur. Radiographic records were reviewed to identify patients in this cohort who had available scout views from CT imaging prior to official diagnosis of the bony lesion or fracture. CT scout images were assessed by two independent reviewers to identify any pathologic lesions, and radiographic reports were reviewed to identify if the lesions were noted by radiology at the time of the initial scan interpretation. One hundred and forty-four patients were identified, and thirty-nine had an available scout CT image prior to official diagnosis of the lesion. Twenty-five patients (64.1%) had lesions identified by authors on scout CT versus only 9 (23.1%) who had lesions that were documented in the initial CT radiologic report. There was a total of 29 lesions identified by the study authors on scout CT, and 19 (65.5%) were not reported in the initial radiographic interpretation with an average interval between observation by authors and official diagnosis of 202 days. Of the impending fractures, three patients (16.7%) went on to complete fracture prior to referral to orthopedics with an average interval between these missed lesions on scout CT and their presentation with fracture of 68 days. This study advocates for the careful review of all scout CT imaging as an essential part of the work up for metastatic disease and encourages all practitioners to utilize this screening tool for the identification of pathologic bony lesions which may help expedite early treatment to reduce patient morbidity.

## 1. Introduction

Pathologic bone lesions, specifically metastatic lesions to bone, can lead to significant disability and morbidity. Bone is the third most common site of cancer metastasis [1], most frequently arising from lung, prostate, and breast, and occurring in 7%, 7%, and 3% of all patients with those respective diagnoses [2]. The prognosis after bone metastasis is poor with a 1-year survival of 51% in breast cancer, 35% in prostate cancer, 29% in renal cell cancer, 13% in bladder cancer, and 10% in lung cancer [2]. Metastasis is also a major cause of morbidity in the form of severe pain, impaired mobility, pathologic fracture, bone marrow aplasia, and symptomatic hypercalcemia [3]. Treatment modalities for bone lesions include pain management, radiation therapy, systemic therapy, and surgery. The decision algorithm for treatment is complicated and often multidisciplinary; however, early detection and management can improve the quality of life and functional outcomes [4]. Pathologic fracture, occurring in approximately 10–30% of all patients with bone metastasis, is a significant sequela affecting patient morbidity and the quality of life. Bone scintigraphy, plain radiographs, computed tomography (CT), magnetic resonance imaging (MRI), and positron emission tomography (PET) scans are all useful tools in the diagnosis of pathologic bone lesions [1].

CT imaging is one of the most widely used techniques for both the initial work up and subsequent surveillance of visceral disease, and when obtained in patients with metastatic disease can be extremely valuable for the evaluation of osseous structures [5–9]. However, there are limitations to its musculoskeletal application. One of the most problematic is that CT scans of the chest, abdomen, and pelvis often do not include long bones in the field of study [10]. Therefore, when used as the sole surveillance tool, CT scans may not adequately show lesions that would be more easily seen with dedicated radiographic imaging [10, 11]. The scout CT view completed at the time of CT imaging displays a larger anatomic field of by showing adjacent anatomy not included on tomographic images [10]. Reviewing the scout CT does not add a significant amount of time to image assessment [12]. Compared to conventional radiographs, scout CT imaging is not subject to parallax distortion present in cone-beam imaging geometry which can reduce accuracy in the diagnosis of fractures [11]. Additionally, the CT scout view alone has up to 300 times lower radiation burden to the thyroid than CT imaging [13]. The scout CT image is integrated into all CT imaging and is not performed independently. Therefore, a careful review of the scout CT view may add diagnostic information without adding any additional radiation to the patient or time to clinical evaluation.

The purpose of this study is to evaluate the benefit of reviewing scout CT images, obtained for routine oncologic surveillance, for the early identification of pathologic bony lesions.

## 2. Materials and Methods

After Institutional Review Board approval, a retrospective review was conducted to identify all patients who previously underwent surgical treatment for pathologic bone lesions or fractures of the humerus or femur by two fellowship-trained orthopedic oncology surgeons at a tertiary care institution between 2009 and 2019. Radiographic records were, then, reviewed to identify patients in this cohort who had available scout views from CT imaging of the chest, abdomen, and/or pelvis prior to official diagnosis of the bony lesion or fracture. The time of official diagnosis was defined as the first documented evidence of a pathologic lesion on any form of imaging. All patients included in the study were referred to orthopedic oncology after presenting to primary care, oncology, or the emergency department complaining of pain where imaging was obtained showing a lesion in the involved extremity. Lack of CT scout imaging available prior to the initial diagnosis of the bony lesion, scout images that did not include an adequate anatomic view of the humerus and/or femur, and CT images without a dictation by a radiologist at time of the study were excluded.

Demographic, clinical, and radiographic data were, then, collected on all patients with available scout CT images. Demographic characteristics included the age, sex, race, body mass index (BMI), comorbid disease, and smoking status. Clinical characteristics included the cancer type, site of involvement, fracture and/or lesion characteristics, and treatment modality. Follow-up data included the date of official diagnosis, date of surgery, and date of most recent follow-up. All available CT scout images were assessed by two independent reviewers to identify any pathologic lesions, and radiographic reports were reviewed to identify if the lesions were noted by radiology at the time of the initial scan interpretation. Bony sites of involvement were categorized as proximal third, diaphyseal, and distal third of the involved humerus or femur. Lesions were classified as impending fractures based upon the following criteria: a lesion size greater than one-third the diameter of the bone, lesions located in the femoral neck, intertrochanteric or subtrochanteric regions of the femur, or the proximal humerus, and/or mutual agreement by the senior authors that the lesion could lead to pathologic fracture based on several factors including the tumor subtype and remaining bone stock. Lesions were classified as complete fractures when a distinct fracture line was present in at least one cortex.

Descriptive statistics were used for overall cohort analysis. Comparative statistical analyses were performed using mean values and standard deviations for continuous variables and frequencies and percentages for categorical variables. Cohorts of patients with unobserved lesions and patients without unobserved lesions were compared using Student's *T*-tests for continuous variables and Fisher Exact tests for categorical variables. All comparative analyses were two-tailed and used an alpha of 0.05.

## 3. Results

**Figure 1:**
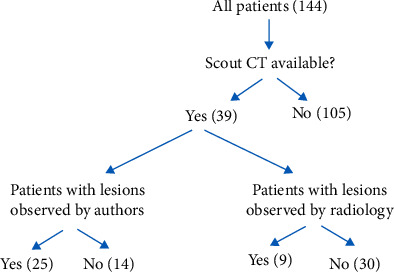
Illustration of the number of patients included and excluded from the initial patient population. The number of patients with lesions observed by the authors on scout CT and the number of patients with lesions documented on the initial radiographic dictation are shown. CT, computed tomography.

One hundred and forty-four patients who underwent surgical treatment for pathologic lesions or fractures were identified. Thirty-nine patients had available scout CT images that met the inclusion criteria and were subsequently included in this analysis (Figure 1). The mean age was 61 years (range, 38–79), and 23 patients (66.7%) were female. The majority of patients were Caucasian (92.3%) with an average BMI of 27.0 (range, 16.6–71.2). There were 24 patients (61.5%) with a history of smoking. Comorbidities included cardiovascular disease (61.5%), chronic obstructive pulmonary disease (15.4%), diabetes mellitus (15.4%), and hypothyroidism (15.4%). Breast cancer was the most common primary oncologic diagnosis with a total of 16 patients (41.0%). Thirty-four patients (87.2%) had observed lesions in the femur, and 5 (12.8%) patients had lesions in the humerus (Table 1).

Of the 39 included patients, 25 patients (64.1%) had lesions identified by the study authors on scout CT versus only 9 patients (23.1%) who had lesions that were documented in the official CT radiologic report. There were a total of 29 lesions identified by the study authors on scout CT compared to only 10 total lesions documented in the official radiologic report (Figure 1; Table 2). Patients with missed lesions and patients without missed lesions were similar in all demographic data with the exception of age. Patients who had lesions observed by authors on scout CT that were not reported on initial radiographic dictation were significantly younger than patients with lesions identified by radiology (55 years vs. 65 years; *p*=0.006) (Table 1). One patient had both a lesion identified by authors on scout CT that was not reported on initial radiographic dictation and a separate lesion that was identified on the initial dictation; thus, this patient was included in analysis of both groups.

Overall, there were nineteen lesions (65.5%) observed by the authors on scout CT that were not reported on the initial radiographic interpretation. The average time interval between observation of these nineteen lesions by the authors on scout CT and their official diagnosis was 202 days (3 to 1752) (Table 3). Of these, seventeen were identified in the femur and two were identified in the humerus (Table 2). One of the nineteen lesions observed was a complete pathologic fracture, and eighteen of the lesions observed were impending pathologic fractures. The one patient with a complete pathologic fracture of the left proximal femur seen on scout CT was a unique case. This patient underwent CT of the chest for the evaluation of known metastatic breast cancer, during which time the patient was nearly asymptomatic and ambulatory with only mild hip pain. At the next routine appointment, oncology obtained radiographs due to mild left hip pain, the complete pathologic fracture was discovered, and the patient was referred to orthopedic surgery for fixation.

**Figure 2:**
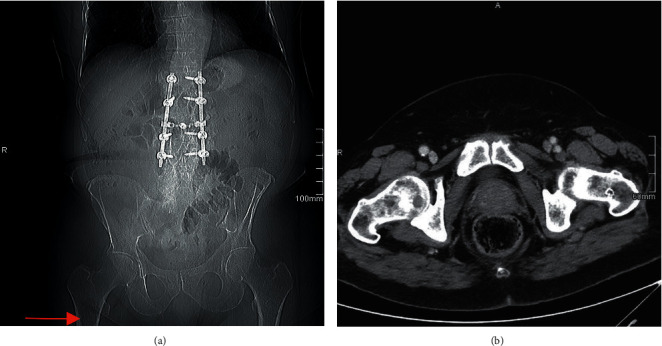
Imaging of a patient with an unobserved pathologic lesion who went on to complete fracture. (a) Routine surveillance scout CT film demonstrating a pathologic lesion with an impending fracture in the right subtrochanteric femur that was not documented in the initial radiographic report. (b) An axial CT view in which the lesion identified by authors in the scout images is not well visualized and was not described in the formal radiographic report. These are of a 76-year-old patient with known metastatic renal cell carcinoma being managed on pazopanib chemotherapy per oncologic specialists.

**Figure 3:**
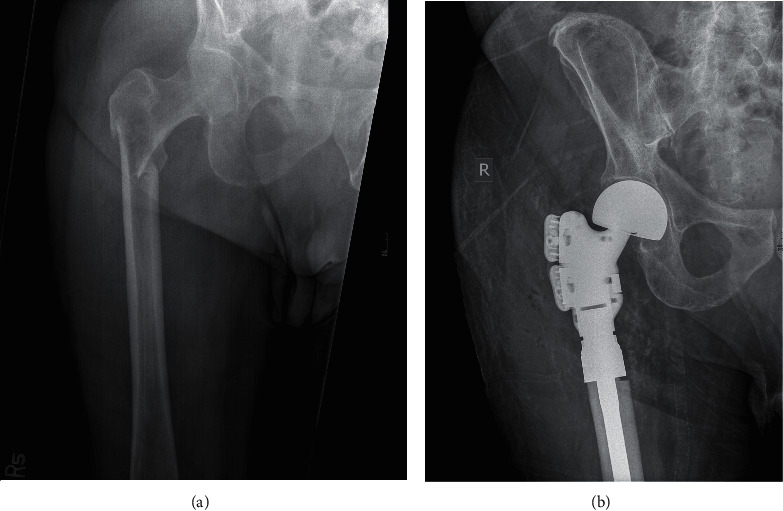
Injury and postoperative radiographs of the pathologic subtrochanteric femur fracture in the patient described in Figure 2. Twelve days after the routine surveillance CT, the patient sustained a pathologic subtrochanteric femur fracture after a fall from a standing height (a). He subsequently underwent proximal femoral replacement by an oncology fellowship-trained surgeon (b).

Of the 18 impending fractures, three patients (16.7%) went on to complete fracture prior to referral to orthopedics, an example of which be seen in Figures 2 and 3. The average time interval between these missed lesions on scout CT and their presentation with fracture was 68 days (13 to 160) (Table 3). Two of the impending fractures that progressed to complete fracture underwent intramedullary fixation, and one underwent resection and arthroplasty. Fourteen impending pathologic fractures subsequently underwent prophylactic intramedullary fixation, and one was treated nonoperatively. The one complete pathologic fracture underwent intramedullary fixation. Two patients had postoperative complications, both of which were wound infections requiring reoperation. One patient underwent debridement of the infected femoral intramedullary nail surgical site, and one patient underwent debridement of the infected hip arthroplasty surgical site followed by hindquarter amputation of the leg (Table 3).

Of the 10 lesions that were reported by radiology on the initial CT dictation, all were referred to orthopedics after identification and presentation with pain. One lesion underwent intramedullary fixation of a complete pathologic fracture that occurred nine days after the CT imaging, just prior to orthopedic evaluation. Eight impending pathologic fractures underwent prophylactic intramedullary fixation. One lesion was seen in a patient with bilateral impending pathologic fractures, and this contralateral side was treated nonoperatively.

## 4. Discussion

To our knowledge, there is no literature to support the review of scout CT imaging for the diagnosis of pathologic bony lesions and/or fractures. Scout CT imaging has been shown to be a valuable diagnostic tool; however, not all hospital systems have an institutional policy to routinely review scout images despite the suggested moral and legal implications [14]. Thus, there is potential for clinically important skeletal findings to go unrecognized. Of the patients in our study who had a scout CT scan prior to an impending or complete pathologic fracture requiring fixation, 64% (25/39) had lesions visible on the CT scout. However, only 23% (9/30) of the patients had lesions identified in the initial radiographic report. Furthermore, 65.5% (10/29) of lesions that were observed by authors on scout CT were not reported on the initial radiographic dictation. These lesions were identified on scout CT at an average of 202 days prior to their official diagnosis. Thus, a significant number of patients with clinically relevant bony pathology could have possibly received an earlier diagnosis and subsequent treatment in the form of prophylactic surgery or radiation therapy through a careful review of scout CT imaging. This is most clearly demonstrated by the three patients (16.7%) with an impending fracture observed by the authors that went on to complete fracture prior to referral to orthopedics. These three impending pathologic fractures were identified on scout CT at an average of 68 days prior to their presentation with complete fracture. While this would not be guaranteed, earlier identification of these lesions with timely stabilization could have prevented complete pathologic fracture in this already fragile patient population. Additionally, patients who had lesions observed by authors on scout CT that were not reported on initial radiographic dictation were significantly younger than patients with lesions identified by radiology (55 years vs. 65 years; *p*=0.006). As metastatic lesions are typically seen in an aging population, this trend could represent a lesser degree of suspicion for the identification of metastatic lesions in younger patients. A majority of patients in our study were female (66.7%) and had a history of smoking (61.5%), which is reflective of the most common sites of primary disease observed, the breast (41%) and the lung (17.9%). When using scout CT for screening purposes, patients with these comorbidities and primary cancer types should raise concern for existing pathologic lesions. Additionally, a majority of the lesions identified were located in the proximal femur (87.2%) compared to the proximal humerus (12.8%). Lesions of the proximal femur often have a lower threshold for prophylactic stabilization given the weight bearing load through the lower extremities which could explain this trend. All lesions that were identified by the authors on scout CT were either impending or complete pathologic fractures. None of the lesions observed were incidental findings without an impending fracture concern. We believe this is secondary to the resolution of scout views only allowing significant lesions to be identified, coupled with a field of view that includes the axial and proximal appendicular skeleton (areas more prone to weight bearing and stress). This may suggest that lesions observed on scout CT are at risk for pathologic fracture.

While the existing studies have shown a propensity for unidentified clinical findings on CT imaging when compared to scout CT views, none are specific to pathologic bone lesions of the appendicular skeleton. Additionally, some studies utilized only single-view lateral scout CT images, rather than a two-view scout CT, which could limit their detection capabilities [12, 13, 15]. A study by Johnson et al. retrospectively reviewed 2,032 scout CT images for pathologic findings and compared their findings to the initial radiologist dictation for the associated CT scan. They included patients with both single-view and two-view scout imaging. The authors found that pathologic findings apparent on scout CT views were not commented on the interpretation of the CT scan in approximately 1% of cases, with the most commonly missed finding being cardiomegaly. While this rate seemed low, if extrapolated by the large number of CT scans ordered across the country per year, it translates into a significant number of patients with unobserved clinically relevant findings [10]. This study included both visceral and musculoskeletal pathology in their review; thus, its findings cannot be directly translated to an orthopedic oncologic patient population. A retrospective study conducted by Bazzocchi et al. showed an even higher rate of unobserved clinical findings on CT scan interpretation when compared with a lateral-only scout CT review. These authors identified osteoporotic vertebral fractures in 14% of their patients on scout CT, 73% of which were not diagnosed by radiologists on CT dictation [15]. While this study presents an analysis of unidentified axial fractures, it does not include the incidence of oncologic bone lesions in their results. Scout CT imaging does have its limitations, as the image itself lacks the resolution and quality offered by traditional CT. However, several studies have shown its usefulness in the detection of orthopedic conditions. In a prospective study by Theocharopoulos et al. comparing helical CT and lateral-only scout CT imaging in the diagnosis of low severity cervical spine fractures, scout CT was shown to be 70% sensitive and 100% specific [13]. In another retrospective study by Bazzocchi et al., lateral-only scout CT was shown to have sensitivity and specificity approaching 99% in the detection of thoracolumbar vertebral compression fractures [12]. The abovementioned studies demonstrate both the utility of scout CT for the identification of potentially missed clinical findings and their high specificity for the visualization of bony pathology.

Prophylactic fixation of impending pathologic fractures has clinical and economic benefits compared to acute fracture treatment. Blank et al. found that the total cost was higher in patients treated with complete pathologic fractures versus impending fractures by an estimated 21,000 USD (U.S. Dollar). In addition, the mean length of stay was eight days after complete fracture fixation versus only four days for an impending fracture prophylactic fixation [16]. Mosher et al. used The Nationwide Inpatient Sample database from 2002-2014 and found that prophylactic fixation was associated with decreased in-hospital mortality, acute renal failure, anemia, and peri- and postoperative blood transfusions. Similarly, prophylactic fixation had a decreased length of stay and total charges (−3,405 USD, *p*=0.001) compared to acute fracture treatment [17]. A 2019 article by El Abiad et al. used the American College of Surgeons National Surgical Quality Improvement Program database to compare 30-day postoperative outcomes in prophylactic versus postfracture stabilization for metastatic lesions of the long bones and found lower risk of discharge to a facility other than home (OR 0.48, *p*=0.02), shorter length of hospital stay (IRR = 0.86, *p*=0.01), and lower risk of major medical complications (OR = 0.64, *p* < 0.01) compared with postfracture stabilization [18]. While the literature supports the early treatment of impending fractures to lower the risk of complications, shorten hospital stays, and lower overall health costs, identifying at-risk lesions is challenging. Our results support the use of routine surveillance scout CT images as one tool to help identify high-risk pathologic lesions.

This report is not without limitations. This was a retrospective review of patients who were known to have pathologic lesions or fractures. There is inherent selection bias in retrospectively reviewing scout CT films in the area of a later diagnosed fracture or lesion. Additionally, there is no standardization at our institution for reviewing scout CT imaging, and the review may not have been included in the formal radiographic report. Another potential limitation of our study is dependent on who is reviewing the CT imaging and why it was ordered. CT scans in this patient population were typically obtained for the evaluation of visceral disease, and osseous structures may not have been a primary focus of interpretation. These CT scans are intricate, and identifying every disease focus and possible treatment effect is daunting and likely not feasible. In addition, the field of view on scout imaging of the chest and abdomen-pelvis is often limited to the surgical neck of the humerus and the subtrochanteric region of the femur. In our study, the majority of lesions identified were located in the proximal humerus and proximal femur, which indicates the low sensitivity of scout CT for the evaluation of more distal lesions. Also, identifying humeral lesions on scout is highly variable as most chest CT imaging is completed with the patient's arms overhead. Therefore, while scout CT may be a good screening tool, it does not define lesions, as well as dedicated radiographs, and should not be used as a true substitute. We do not suggest that patients should have their operative plan based on scout imaging; rather, we argue that their detection can lead to more dedicated imaging and possibly prevent catastrophic skeletal failure. Medical oncologists who are reviewing surveillance imaging also may not be specifically trained in radiographic review to identify lesions and/or impending fractures, and orthopedic oncologists are generally involved after diagnosis. However, if scout CT imaging is routinely reviewed, the orthopedic surgeon may have the chance to be involved earlier and help to prevent skeletal related events such as fracture. Another limitation of our study is the lack of pain score analysis in the determination of impending pathologic fractures, as Mirels' criteria describes the important role of pain for the identification of impending fractures [19]. All patients in our study with a scout CT prior to official diagnosis of a lesion were subsequently referred to orthopedics due to pain in the involved extremity or had a complete fracture at a later date. However, our retrospective study highlights the radiographic evaluation of pathologic lesions and suggests that earlier identification could lead to an evaluation prior to the onset of pain and/or fracture. The sample size in our study only included patients treated by our two fellowship-trained orthopedic oncology surgeons and may have excluded patients treated during off-hours. Finally, while our patient population was limited to those who were treated by an orthopedic oncologist, there are many patients with pathologic bony lesions who are treated with radiation therapy alone and are never seen by an orthopedist. We recommend future studies that would include all patients with a pathologic bony lesion, regardless of orthopedic involvement.

## 5. Conclusions

Scout images from routine oncologic surveillance CT are a valuable tool to aid in the screening and diagnosis of pathologic long bone lesions that may otherwise not be evaluated by formal CT imaging. We advocate for the careful review of all scout CT images as an essential part of the work up for metastatic disease and encourage all practitioners to utilize this screening tool for the identification of pathologic bony lesions prone to fracture which may help expedite early treatment to reduce patient morbidity.

## Figures and Tables

**Table 1:** Demographic characteristics of included patients, patients with unobserved lesions, and patients without unobserved lesions.

Demographics	All patients (*n* = 39)	Patients with missed lesions (*n* = 17)	Patients without missed lesions (*n* = 23)	*p* value
Age in years (mean) (range)	61 (38, 79)	55 (38, 76)	65 (44, 79)	0.006^*∗*^
Gender				
Male (*n*) (%)	13 (33.3%)	6	7	1.0
Female (*n*) (%)	26 (66.7%)	11	16	1.0
Ethnicity				
Caucasian (*n*) (%)	36 (92.3%)	14	23	0.069
African American (*n*) (%)	3 (7.7%)	3	0	0.069
BMI (mean) (range)	27.0 (16.6, 71.2)	28.0 (16.6, 71.2)	26.1 (19.4, 36.5)	0.636
History of smoking (*n*) (%)	24 (61.5%)	9	15	0.522
Comorbidities				
Cardiovascular disease (*n*) (%)	24 (61.5%)	9	16	0.336
Chronic kidney disease(*n*) (%)	3 (7.7%)	2	1	0.565
Chronic obstructive pulmonary disease (*n*) (%)	6 (15.4%)	1	5	0.216
Diabetes mellitus (*n*) (%)	6 (15.4%)	2	5	0.677
Rheumatoid arthritis (*n*) (%)	1 (2.6%)	1	0	0.425
Hypothyroidism (*n*) (%)	6 (15.4%)	1	6	0.205
Cancer type				
Breast (*n*) (%)	16 (41.0%)	8	9	0.750
Lung (*n*) (%)	7 (17.9%)	2	5	0.677
Prostate (*n*) (%)	4 (10.3%)	0	4	0.123
Renal (*n*) (%)	4 (10.3%)	2	2	1.0
Multiple myeloma (*n*) (%)	3 (7.7%)	1	2	1.0
Colon (*n*) (%)	1 (2.6%)	1	0	0.425
Hepatocellular (*n*) (%)	1 (2.6%)	1	0	0.425
Melanoma (*n*) (%)	1 (2.6%)	1	0	0.425
Leiomyosarcoma (*n*) (%)	1 (2.6%)	1	0	0.425
Unknown (*n*) (%)	1 (2.6%)	0	1	1.0
Site of lesion				
Femur (*n*) (%)	34 (87.2%)	15	19	1.0
Humerus (*n*) (%)	5 (12.8%)	2	4	1.0

One patient had both a lesion identified by authors on scout CT that was not reported on initial radiographic dictation and a separate lesion that was identified on the initial dictation; thus, this patient was included in analysis of both groups. BMI, body mass index; CT, computed tomography.

**Table 2:** Comparison of the observed findings by the study authors between scout CT and radiology on the initial radiographic dictation.

Population	Authors (*n*)	Radiology (*n*)
Patients with available scout CT images	39	39
Number of patients with lesions identified on scout CT	25	9
Femur	21	7
Humerus	4	2
Total number of lesions identified on scout CT	29	10
Femur	23	9
Humerus	6	1

One patient had both a lesion identified by authors on scout CT that was not reported on initial radiographic dictation and a separate lesion that was identified on the initial dictation; thus, this patient was included in analysis of both groups. CT, computed tomography.

**Table 3:** Description of lesions observed by the study authors on scout CT that were not included on the initial radiographic dictation.

Location	Pathology	Type of lesion	Operative management	Time from scout CT to diagnosis (days)	Go on to complete fracture?	Time from scout CT to fracture (days)
Humerus-proximal	Breast	Impending	IMN	86		
Humerus-diaphyseal	Leiomyosarcoma	Impending	IMN	160	Yes	160
Femur-proximal	Breast	Complete	IMN	3		
Femur-diaphyseal	Breast	Impending	IMN	31	Yes	31
Femur-proximal	Renal	Impending	Arthroplasty	13	Yes	13
Femur-proximal	Lung	Impending	IMN	14		
Femur-proximal	Melanoma	Impending	IMN	808		
Femur-proximal	Breast	Impending	IMN	7		
Femur-proximal	Breast	Impending	IMN	146		
Femur-proximal	Breast	Impending	Observation	146		
Femur-proximal	Lung	Impending	IMN	17		
Femur-proximal	Breast	Impending	IMN	172		
Femur-proximal	Breast	Impending	IMN	172		
Femur-proximal	Colon	Impending	IMN	28		
Femur-proximal	Myeloma	Impending	IMN	51		
Femur-proximal	Breast	Impending	IMN	1752		
Femur-proximal	Breast	Impending	IMN	165		
Femur-diaphyseal	Renal	Impending	IMN	41		
Femur-proximal	Hepatocellular	Impending	IMN	26		
					Average: 202	Average: 68
					Range: 3, 1752	Range: 13, 160

IMN, intramedullary nailing.

## Data Availability

The data used to support the findings of this study are available from the corresponding author upon request in order to protect patient privacy.
